# Lecture on Dulcamara

**Published:** 1888-02

**Authors:** A. McNeil

**Affiliations:** San Francisco


### LECTURE ON DULCAMARA.

Dr. A. McNeil, San Francisco.

Dulcamara is an antipsoric and also an anti-sycotic, though in less degree. Its most distinguishing characteristics are its aggravations, which are thus tersely described by Dr. Guernsey: "All the symptoms are aggravated when the weather suddenly becomes colder, especially if it be damp; not so much so that the child (patient) takes cold, but that the morbid condition of the child is such as to be influenced by the atmospheric change. This is on the same principle as the aggravation of Rhododendron, which occurs when there is a wind storm even though the patient be warm and in bed." In this and many other symptoms our remedy has a striking resemblance to Rhus tox., although the latter is not an antipsoric. Both have relief from motion and are aggravated from rest; both are aggravated by cold applications, particularly when wet.

But the Dulcamara patient is irritable, the Rhus patient is anxious and depressed; with Dulcamara the swollen glands are painless; with Rhus, painful; with Dulcamara the menses are late, scanty, and generally of too short duration; while with Rhus, they are too soon, profuse, and of long duration; with Dulcamara the patient is worse before midnight; with Rhus he is worse from evening till morning. With Dulcamara, as with all other drugs, the mental symptoms are the most characteristic. The Dulcamara patient is inclined to scold (Mosch.) without being angry. He has an inability to speak. Knows what he has to say but cannot find the right word (Sulph.); if he is told the word he can say it easily. He has great restlessness and impatience. He has delirium at night during the pain and the fever (hot stage). Please observe the peculiarities of the vertigo of this drug. On awaking in the morning giddy and dizzy (Lachesis), dark before the eyes, and weakness. While our remedy is not a routine one for headache, yet it will cure certain ones when nothing else will. A boring pain in the head from within outward, aggravated before midnight and by lying quietly. Sensation of enlargement of the cerebellum. On the scalp thick crusts form, while the hair falls out. In the eyes we find inflammation caused by catching cold in damp, cold places, and a twitching of the eyelids and lips in cold air. Coming to the ears, we have hardness of hearing, with roaring in the ears and rush of blood to the head. There is a bleeding of the nose cured by Dulcamara, of a very bright red blood, accompanied by a pressure at the root of the nose (which Ruta has, but not the same kind of blood). In the face we have a peculiar alternation of symptoms. After disappearance of tetters, face ache and violent asthma. These alternations of disease manifestations are very important, and are evidence of the truth of Hahnemann's psoric theory. The Dulcamara patient has sometimes a circumscribed redness of the cheeks with paleness of the face (which Lyc. and Phos. also have). In common with Mercurius Dulcamara has dryness of the tongue (subjective) with much thirst and increased flow of saliva, and like Mercurius, the tongue is swollen so that he cannot be understood, yet he talks continually. Sometimes a person takes cold so that his tongue becomes partially paralyzed and the jaws stiff; Dulcamara is his remedy.

In the fevers of this drug there is violent thirst for cold liquids (also Aconite, Ars., etc.). In intermittents he has violent hunger after the hot stage. (With China the thirst is in all stages and during the apyrexia.) In the stomach and abdomen our patient is troubled with empty eructations, with throbbings as from disgust. (With Ant-tart. and Puls. we have throbbing or pulsation, but not with the same concomitants.) The Dulc. patient is also troubled with frequent eructations while eating. (With Argent-n. it is after eating; with Iodium it is from morning till evening; with Kali-carb. it alternates with a weak, empty feeling at the epigastrium.) He has a sensation of retraction at the epigastrium with burning. (Also Plumb: With colicky pains, is Podoph.) He is tormented with a violent pinching in the abdomen, as if a worm were crawling up and down in it, and was gnawing and pinching the parts. He vomits whitish, tough mucus.

Dulcamara is sometimes indicated in discharges arising from wet, cold weather, and from cold, damp nights, while the days are hot. They are usually worse at nights and of various colors, but often mucous in character and are accompanied by colic and thirst. Catarrhs of the bladder from damp, cold weather always remember Dulcamara; for instance, in catarrhal ischuria in children from wading in water with bare feet, when the discharge is a milky, mucous urine. The urine on standing and becoming cooled, has an oily consistence with a jelly-like sediment intermixed with specks of blood. Sometimes the urine sticks and has a mucous sediment. In the diseases of the female generative organs Dulcamara plays an important part, particularly in suppression of the menses, or of the lochia from damp, cold weather, or in cases of threatened miscarriage, or loss of the mother's milk from the same cause. When a rash (Kali-carb., a nettle rash) appears on the skin before the menses. (With itching between the shoulder-blades, Carbo-veg.; on the forehead, Sarsap.) She may have her menses too late and lasting too short a time, with a watery discharge.

In children inclined to salivation (also Merc.), diarrhœas, and eruptions. In the back pains and also those in the limbs we find a striking resemblance between Dulcamara and Rhus. We have in Dulcamara pain in the small of the back, as after stooping too long a time, and still more so after taking cold; then we have neck stiff, and the back painful, the loins lame. And again, during rest drawing from the loins through to the thigh, which becomes a sticking on moving, and is ameliorated on rising. In paralysis this resemblance continues to be observed; Dulcamara has paralysis of the arms, which are very cold and are worse at rest. Dulcamara is a good remedy for warts on the hands, also in exostoses on the upper part of the right tibia with bluish-red spots, suppurating lumps. Dulcamara cures formication as from ants in the feet. (Secale has a similar tingling in the toes.)

On the skin the action of Dulcamara is marked. It has eruptions with reddish margins, and which become painful, but not itchy, from cold weather. Here again the bad effects of cold are seen; every time she takes cold urticaria or some other cutaneous affection appears; also complaints from retrogression of eruptions from damp, cold air; also dropsical affections from suppression of sweat by damp, cold air. Eczema oozing a watery fluid (also Graph.), bleeding after scratching. Small boils on places formerly bruised. It is a prominent remedy in hives, and is indicated in that form which itches much; also burning after scratching; it increases in warmth and disappears after taking cold, with gastric fever.

Dulcamara and Baryta carb. are complimentary.

